# Response‐adapted treatment with rituximab, bendamustine, mitoxantrone, and dexamethasone followed by rituximab maintenance in patients with relapsed or refractory follicular lymphoma after first‐line immunochemotherapy: Results of the RBMDGELTAMO08 phase II trial

**DOI:** 10.1002/cam4.2555

**Published:** 2019-10-01

**Authors:** Francisco‐Javier Peñalver, José‐Antonio Márquez, Soledad Durán, Pilar Giraldo, Alejandro Martín, Carlos Montalbán, Juan‐Manuel Sancho, María‐José Ramírez, María‐José Terol, Francisco‐Javier Capote, Antonio Gutiérrez, Blanca Sánchez, Andrés López, Antonio Salar, Gil Rodríguez‐Caravaca, Miguel Canales, María‐Dolores Caballero, on behalf of GELTAMO (The Spanish Lymphoma Cooperative Group)

**Affiliations:** ^1^ Hospital Universitario Fundación Alcorcón Alcorcón Madrid Spain; ^2^ Hospital de Basurto Vizcaya Spain; ^3^ Complejo Hospitalario de Jaén Jaén Spain; ^4^ Hospital Universitario Miguel Servet Zaragoza Spain; ^5^ Hospital Universitario de Salamanca IBSAL CIBERONC Salamanca Spain; ^6^ Hospital Universitario Ramón y Cajal Madrid Spain; ^7^ ICO‐IJC Hospital Germans Trias i Pujol Badalona Barcelona Spain; ^8^ Hospital de Especialidades de Jerez de la Frontera Cádiz Spain; ^9^ Hospital Clínico Universitario de Valencia Valencia Spain; ^10^ Hospital Puerta del Mar Cádiz Spain; ^11^ Hospital Universitario Son Dureta Palma de Mallorca Spain; ^12^ Hospital del Mar Barcelona Spain; ^13^ Hospital Vall d'Hebron Barcelona Spain; ^14^ Universidad Rey Juan Carlos Madrid Spain; ^15^ Hospital Universitario La Paz Madrid Spain

**Keywords:** bendamustine, follicular lymphoma, immunochemotherapy, refractory, relapsed

## Abstract

**Background:**

Consensus is lacking regarding the optimal salvage therapy for patients with follicular lymphoma who relapse after or are refractory to immunochemotherapy.

**Methods:**

This phase II trial evaluated the efficacy and safety of response‐adapted therapy with rituximab, bendamustine, mitoxantrone, and dexamethasone (RBMD) in follicular lymphoma patients who relapsed after or were refractory to first‐line immunochemotherapy. Sixty patients received three treatment cycles, and depending on their response received an additional one (complete/unconfirmed complete response) or three (partial response) cycles. Patients who responded to induction received rituximab maintenance therapy for 2 years.

**Results:**

Thirty‐three (55%) and 42 (70%) patients achieved complete/unconfirmed complete response after three cycles and on completing induction therapy (4‐6 cycles), respectively (final overall response rate, 88.3%). Median progression‐free survival was 56.4 months (median follow‐up, 28.3 months; 95% CI, 15.6‐51.2). Overall survival was not reached. Progression‐free survival did not differ between patients who received four vs six cycles (*P* = .6665), nor between patients who did/did not receive rituximab maintenance after first‐line therapy (*P* = .5790). Median progression‐free survival in the 10 refractory patients was 25.5 months (95% CI, 0.6‐N/A) and was longer in patients who had shown progression of disease after 24 months of first‐line therapy (median, 56.4 months; 95% CI, 19.8‐56.4) than in those who showed early progression (median, 42.31 months; 95% CI, 24.41–NA) (*P* = .4258). Thirty‐six (60%) patients had grade 3/4 neutropenia. Grade 3/4 febrile neutropenia and infection were recorded during induction (4/60 [6.7%] and 5/60 [8.3%] patients, respectively) and maintenance (2/43 [4.5%] and 4/43 [9.1%] patients, respectively).

**Conclusions:**

This response‐adapted treatment with RBMD followed by rituximab maintenance is an effective and well‐tolerated salvage treatment for relapsed/refractory follicular lymphoma following first‐line immunochemotherapy.

**Clinical trial registration:**

http://clinicaltrials.gov # NCT01133158.

## Graphical abstract

RBMD using a response‐adapted strategy followed by rituximab maintenance is an effective and safe salvage treatment for relapsed/refractory follicular lymphoma following first‐line immunochemotherapy, thereby improving tolerability without compromising efficacy.

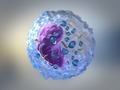

## 1 INTRODUCTION

Follicular lymphoma (FL) is the most common form of indolent lymphoma and the second most frequent subtype of non‐Hodgkin's lymphoma (NHL) diagnosed in the Western hemisphere.^1^, ^2^ Rituximab plus chemotherapy has significantly improved outcomes in patients with newly diagnosed FL and emerged as the standard of care for frontline therapy.^3^, ^4^, ^5^, ^6^, ^7^ Rituximab maintenance after induction therapy has significantly improved progression‐free survival (PFS) in FL patients.^8^ Although FL usually responds to first‐line immunotherapy or immunochemotherapy, the majority of patients relapse.

In the absence of consensus regarding optimal salvage therapy, the selection of treatment for relapsed FL poses a daunting challenge. Moreover, there are limited treatment options for patients with refractory FL. Multiple factors influence the choice of salvage treatment, including prior treatment, duration of prior response, age, comorbidities, and therapeutic goals.^2^ When this trial was designed recommended treatment options included fludarabine‐based regimens combined with rituximab^9^ and R‐CHOP (rituximab, cyclophosphamide, doxorubicin, vincristine, and prednisone).^10^, ^11^

Two phase II trials of bendamustine plus rituximab (BR) as salvage therapy in indolent lymphoma patients reported overall response rates (ORR) in FL subgroups of 93%^12^ and 96%^13^ (median PFS, 23 and 24 months, respectively). In a German prospective randomized phase III trial, BR was more effective than fludarabine plus rituximab (FR) in patients with relapsed indolent lymphoma.^14^

Survival is poor in treatment‐refractory FL patients and those with early progression after first‐line therapy,^15^, ^16^, ^17^ particularly in those previously treated with rituximab.^18^ A phase II trial of the combination of bendamustine, mitoxantrone, and rituximab found that it was effective (ORR, 92%; median PFS, 19 months) and well‐tolerated in patients with relapsed/refractory indolent lymphoma with or without prior rituximab‐containing immunochemotherapy.^19^

While rituximab maintenance has shown beneficial effects in relapsed patients not previously exposed to rituximab in first‐line therapy,^10^ few studies have evaluated rituximab maintenance at the moment of relapse in patients previously exposed to rituximab in first‐line therapy,^10^, ^14^ and available information on the usefulness of second rituximab maintenance therapy is anecdotal.^20^ Data on the effect of rituximab maintenance after treatment with BR in patients with relapsed/refractory indolent lymphoma are limited to the findings of a phase III trial in which a small subgroup of patients (two thirds of cases were rituximab‐naïve) received rituximab maintenance after responding to BR.^14^ Obinutuzumab plus bendamustine followed by obinutuzumab maintenance has shown clinically beneficial effects in rituximab‐refractory patients with indolent NHL.^18^

In this phase II trial, we evaluated the efficacy and safety of salvage therapy with RBMD followed by rituximab maintenance in patients with relapsed/refractory FL after first‐line rituximab‐based immunochemotherapy.

## 2 MATERIALS AND METHODS

### 2.1 Study design and patient eligibility

RBMDGELTAMO08 is a multicenter, phase II trial conducted at 27 Spanish centers by GELTAMO (The Spanish Lymphoma Cooperative Group).

The study population consisted of patients who had relapsed after or were refractory to first‐line rituximab‐based immunochemotherapy, including those who had received rituximab maintenance after first‐line therapy. Patients eligible for inclusion were those aged 18‐75 years who had been previously diagnosed (lymph node or tissue biopsy) by a local pathologist and fulfilled the following criteria: FL grade, 1‐3a; Eastern Cooperative Oncology Group (ECOG) score, ≤2; Ann Arbor stage, I–IV; Follicular Lymphoma International Prognostic Index (FLIPI), 0‐5. Patients to whom any of the following exclusion criteria applied were considered ineligible: previous radiotherapy treatment; relapse after autologous stem cell transplantation; central nervous system involvement; previous or concomitant malignant disease; clinical suspicion or histological confirmation of transformed lymphoma (in the staging bone marrow biopsy performed on each patient or in a new lymph node or tissue biopsy performed prior to inclusion in some patients with clinical suspicion of transformation); previous or active infection with hepatitis B virus, hepatitis C virus, or HIV; other serious immunosuppressive conditions; organ function deficits (liver, kidney, or heart) unrelated to lymphoma.

Refractory patients were defined as those who were nonresponsive (less than partial response) or showed disease progression after at least three immunochemotherapy cycles, during rituximab maintenance, or within less than 6 months of the last immunochemotherapy cycle or the last dose of rituximab maintenance therapy. Relapse was defined as progressive disease beyond 6 months after the last cycle of first‐line immunochemotherapy or the last dose of rituximab maintenance therapy.

The trial was approved by the Institutional Review Boards and the local ethical committees of each participating center. Patients provided written informed consent before inclusion. All procedures were performed according to the principles expressed in the Declaration of Helsinki. This clinical trial is registered at http://clinicaltrials.gov (NCT01133158).

### 2.2 Treatment and procedures

The RBMD regimen was designed based on modifications of two previously described regimens.^19^, ^21^

Patients received the following treatment every 4 weeks: intravenous infusion of 375 mg/m^2^ rituximab on day 1; 90 mg/m^2^ bendamustine on days 1 and 2; 6 mg/m^2^ mitoxantrone on day 1; and 20 mg oral dexamethasone on days 1‐5.

We used a response‐adapted approach to guide treatment intensity at induction. Patients with complete response/unconfirmed complete response (CR/uCR) after three cycles of RBMD received a total of four cycles, whereas those with partial response (PR) received additional three cycles (six cycles in total). Patients with stable disease/progressive disease (SD/PD) after the third or sixth cycle were withdrawn from the trial. Patients who achieved CR/uCR, or PR at the end of induction therapy (after four or six cycles) received rituximab maintenance (375 mg/m^2^) every 12 weeks for 2 years or until progression or unacceptable toxicity.

Participants underwent laboratory and clinical assessment before each treatment cycle, which they received only upon fulfilling the following criteria: absolute neutrophil count (ANC) >1.5 × 10^9^/L; platelet count >75 × 10^9^/L; absence of toxic effects of grade 3 or higher. If grade 3/4 cytopenia was detected, the next cycle was delayed by 7‐14 days, and bendamustine treatment resumed at a reduced dose of 70 mg/m^2^ for all subsequent cycles. If parameters did not return to baseline levels within 14 days, the cycle was delayed by a further 14 days and bendamustine treatment resumed at a further reduced dose of 50 mg/m^2^ for the remaining cycles. If parameters failed to return to baseline levels after the second 14‐day delay, the patient was withdrawn from the trial. Reduction of the bendamustine dose to 70 and 50 mg/m^2^ was accompanied by a reduction in the mitoxantrone dose to 5 and 4 mg/m^2^, respectively. Treatment was discontinued if toxic effects were observed with the 50 mg/m^2^ dose of bendamustine. Rituximab dose reduction was not permitted. Escalation of the bendamustine dose after a dose reduction was not permitted.

Cycles were delayed for a maximum of 4 weeks in patients with grade 3/4 neutropenia. Longer delays resulted in withdrawal from the trial. Treatment was discontinued in cases of grade >3 nonhematologic adverse events (AEs) and grade 4 infections.

Patients with a lymphocyte count <1 × 10^9^/L or a previous history of herpes virus infection received prophylaxis with co‐trimoxazole and acyclovir. Primary prophylaxis with G‐CSF and secondary prophylaxis or treatment in accordance with the American Society of Clinical Oncology Guidelines were permitted.^22^

### 2.3 Study objectives, response assessment, response criteria, and safety

The primary endpoint was CR/uCR after induction therapy (4‐6 cycles). Secondary endpoints were toxicity, the role of rituximab maintenance to prolong the response and delay the next treatment, OS and PFS.

Treatment response was assessed every 3 months during induction and maintenance treatment, every 6 months for the following 2 years, and then annually. Tumor assessment included clinical assessment, physical examination, a routine laboratory test, and a CT scan. In patients in whom bone marrow infiltration was detected at staging, bone marrow biopsy was repeated in subsequent efficacy reassessments until confirmation of complete response in responding patients.

Patients were classified as CR, uCR, PR, SD, or PD according to the criteria of the National Cancer Institute International Working Group.^23^ PFS was defined as the time from the first dose of treatment to progression or tumor‐related death.

Patients who received at least one treatment cycle were included in the toxicity analysis. AEs were monitored throughout the study and graded according to the National Cancer Institute Common Terminology Criteria for AEs (v3.0).^24^

### 2.4 Statistical analysis

Based on previous studies that reported an ORR of 50% after single agent rituximab, we selected a sample size of 60 patients to enable detection of a 20% increase in ORR after treatment with BR with a statistical power >80% (using a two‐tailed analysis with significance set at 95%). Patients without at least one response assessment were considered nonresponders.

Continuous variables are represented as the mean, standard deviation, median, and range, and qualitative variables as absolute and relative frequencies (%) and corresponding 95% confidence interval (95% CI).

A two‐sided 95% exact CI for ORR was calculated using the binomial distribution. The Kaplan‐Meier method was used to estimate median OS and PFS. Fisher's exact test was used for comparisons of independent groups. Significance was set at *P* < .05 for statistical analyses. SAS^®^ 9.4 was used.

## 3 RESULTS

**Figure 1:**
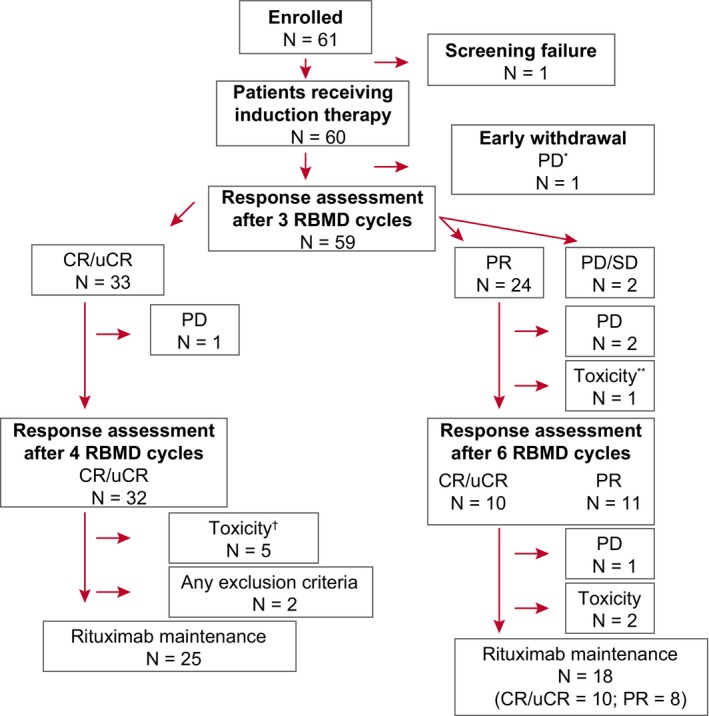
Patient disposition. Patient disposition during induction therapy with RBMD. *1 patient who showed progressive disease during the first RBMD cycle died. **1 patient died due to grade 5 infection (CMV reactivation in the context of influenza A sepsis). ^†^8 patients were withdrawn due to toxicity, including five patients withdrawn due to treatment delays >4 wk. CR, complete response; PD, progressive disease; PR, partial response; RBMD, rituximab, bendamustine, mitoxantrone, and dexamethasone; SD, stable disease; uCR, unconfirmed complete response

Sixty‐one patients were enrolled and assessed. After exclusion of one screen failure, 60 patients (median age, 62.5 years; range, 32‐76) received treatment according to the study protocol (Figure 1). These 60 patients had FL relapsed (n = 50) or refractory (n = 10) to first‐line rituximab‐containing immunotherapy and 43.3% (n = 26) had received rituximab maintenance after first‐line therapy (Table 1, baseline characteristics in the intention‐to‐treat (ITT) analysis). None of the patients had received bendamustine in first‐line therapy.

**Table 1:** Baseline patient demographics and clinical characteristics at enrollment

	N	%
Patients	60	
Age (y)		
<60	26	43
>60	34	57
Sex		
Female	30	50
Male	30	50
B symptoms		
Present	10	17
Absent	50	83
Ann Arbor stage		
I‐II	19	31.5
III‐IV	41	68.5
ECOG‐PS		
0	38	63
1	21	35
2	1	2
FLIPI (number of risk factors)		
Low (0‐1)	30	50
Intermediate (2)	17	28
High (3‐5)	13	22
First‐line treatment		
R‐CHOP	43	71.7
R‐CVP	12	20
R‐FC	5	8.3
Rituximab maintenance after first‐line therapy	26	43.3

Note

Table shows data for 60 patients included in the intention‐to‐treat analysis. B symptoms refer to fever, weight loss >10%, and nights sweats.

Abbreviations: ECOG‐PS, Eastern Cooperative Oncology Group performance status; FLIPI, Follicular International Prognostic Index; R‐CHOP, rituximab, cyclophosphamide, doxorubicin, vincristine, and prednisone; R‐CVP, rituximab, cyclophosphamide, vincristine, and prednisone; R‐FC, rituximab, fludarabine, and cyclophosphamide.

Early response assessment revealed an ORR of 95% (95% CI, 86.1‐99.0): after the third cycle of RBMD, 55% of patients (33/60) achieved CR/uCR (CR‐3 group), 40% (24/60) achieved PR, and 5% (3/60) had PD/SD. Per protocol, 24 patients who achieved PR after three cycles received a total of six treatment cycles (CR‐6 group). Of these, 10 (41.7%) subsequently achieved CR/uCR and 11 (45.8%) maintained PR. On completion of induction therapy (4‐6 cycles), 70% of patients were in CR or uCR and 18.3% in PR, for an ORR of 88.3% (53/60 patients) (Table 2).

**Table 2:** Response to RBMD

	After three cycles (N = 60)			At end of induction therapy (3‐6 cycles) (N = 60)			To R‐maintenance therapy (N = 43)		
	N	%	95% CI	N	%	95% CI	N	%	95% CI
Complete response^a^	33	55	41.61‐67.88	42	70	56.79‐81.85	32	74.5	58.83‐86.48
Partial response	24	40	27.56‐53.46	11	18.3	9.52‐30.44	3	7	1.46‐19.06
Overall response	57	95	86.08‐98.96	53	88.3	77.43‐95.18	35	81.5	66.60‐91.61
Stable disease	1	1.7	0.04‐8.94	1	1.7	0.04‐8.94			
Progressive disease	2	3.3	0.41‐11.53	6	10	4.82‐22.57	8	18.5	8.39‐33.40

Abbreviations: CI, confidence interval; RBMD, rituximab, bendamustine, mitoxantrone, and dexamethasone.

a Complete response includes unconfirmed complete response.

Seventeen patients (28.3%) were withdrawn from the trial before they could begin rituximab maintenance: seven were in PD/SD; two (both in CR) failed to fulfill the inclusion criteria for starting maintenance therapy; and eight (5 in CR/uCR, 3 in PR) due to toxicity. Forty‐three patients (71.7%) began rituximab maintenance: 25 in the CR‐3 group and 18 in the CR‐6 group (10 in CR/uCR, 8 in PR) (Figure 1).

After 2 years of maintenance therapy, 32 patients were in CR/uCR, three in PR, and eight in PD (Table 2). In the CR‐3 subgroup, 65.6% (21/32) of patients maintained their response at the end of maintenance therapy. In the CR‐6 subgroup, two patients in PR achieved CR during maintenance. During the maintenance therapy period, three patients were withdrawn from the trial due to protocol violation/deviations and another three due to toxicity. All six patients left the trial in CR.

**Figure 2:**
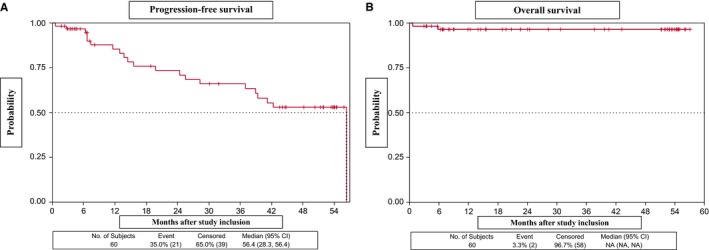
Progression‐free survival (A) and overall survival (B) in patients who received RBMD plus rituximab maintenance therapy

With a median follow‐up of 28.3 months (95% CI, 15.6‐51.2) after completion of rituximab maintenance, eight patients showed PD. Median PFS was 56.4 months (95% CI, 28.3‐56.4), with 2‐ and 4‐year PFS probabilities of 73.4% (95% CI, 57.7‐84.0) and 52.9% (95% CI, 36.6‐66.7), respectively (Figure 2A). Median overall survival (OS) was not reached; the 4‐year OS probability was 96.4% (95% CI, 86.5‐99.1) (Figure 2B).

**Figure 3:**
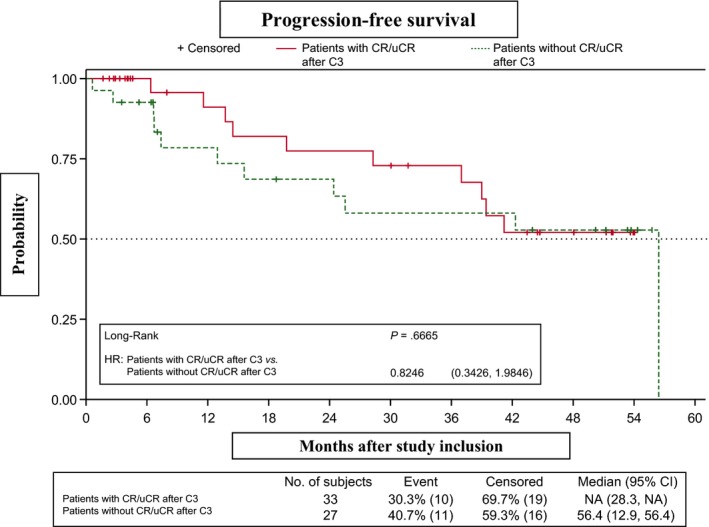
Progression‐free survival in patients with/without complete or unconfirmed complete response (CR/uCR) after three treatment cycles (CR‐3 vs CR‐6)

**Figure 4:**
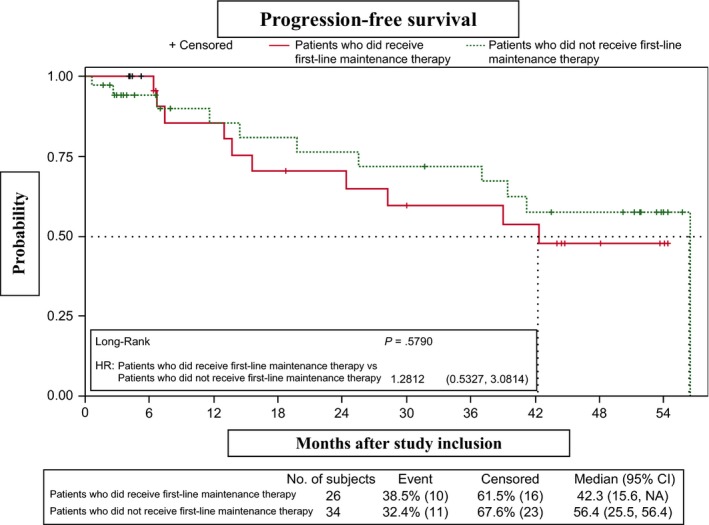
Progression‐free survival in patients who did/did not receive first‐line maintenance therapy

**Figure 5:**
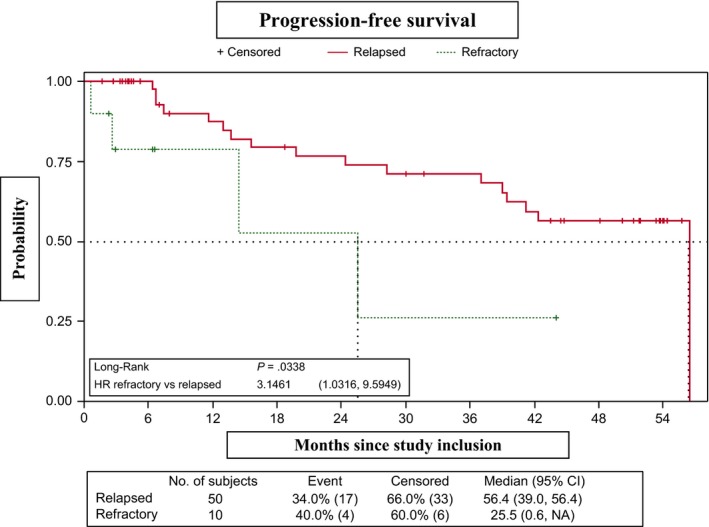
Progression‐free survival (relapsed vs refractory)

**Figure 6:**
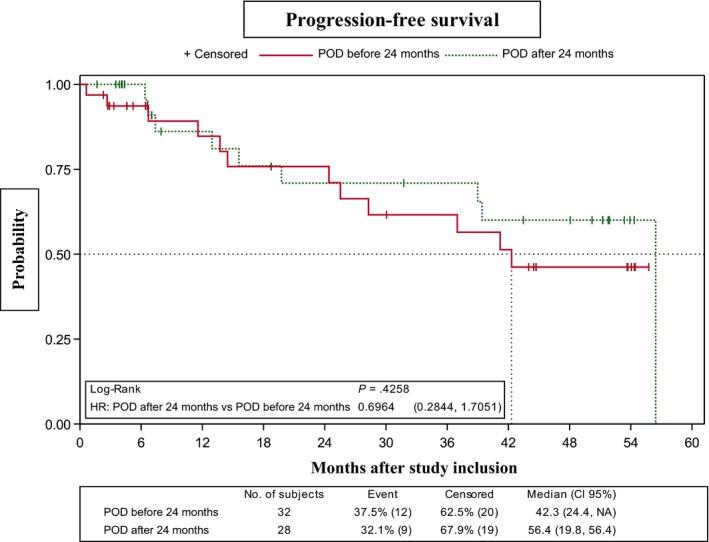
Progression‐free survival according to progression of disease after first‐line immunochemotherapy (before/after 24 mo of treatment) prior to enrollment in the trial

No differences in PFS were observed between the CR‐3 subgroup, which showed an early response to RBMD (four cycles), and the group that received six cycles (*P* = .6665) (Figure 3). Similarly, PFS did not differ significantly between patients who did/did not receive rituximab maintenance in first‐line therapy (median PFS, 42.3 months [95% CI, 15.6–NR] and 56.4 [95% CI, 25.5‐56.4], respectively; *P* = .5790) (Figure 4). However, PFS was significantly lower in patients refractory to first‐line therapy vs relapsed patients (median PFS, 25.5 months [95% CI, 0.6–NR] and 56.4 months [95% CI, 39.0‐56.4], respectively; *P* = .0338). The 2‐year and 3‐year PFS probabilities in the refractory group were 52.5% (95% CI, 8.4‐84.6) and 26.3% (95% CI, 1.1‐67.5), respectively (Figure 5). PFS patients who had shown progression of disease (POD) after 24 months of first‐line therapy had longer (median, 56.4 months [95% CI, 19.8‐56.4]) than those who showed POD after less than 24 months of first‐line therapy (median, 42.31 months [95% CI, 24.41‐NA]), although this difference was not statistically significant (Figure 6).

## 4 SAFETY

All 60 patients who received RBMD were included in the safety analysis.

A total of 275 cycles of RBMD were administered (median and range per patient: 4, 1‐6). The number of chemotherapy cycles per patient was <4 in eight patients (13.3%); four in 27 patients (45%); five in five patients (8.3%); and six in 20 patients (33.3%). The bendamustine dose was reduced in 14 patients, in all cases due to hematological toxicity. Forty‐four of the 275 (16%) cycles were delayed, due to hematological toxicity (28 [10.2%] cycles); nonhematological toxicity (3 [1.1%] cycles); hematological and nonhematological toxicity (3 [1.1%] cycles); or other reasons (10 [3.6%] cycles).

Rituximab maintenance therapy was administered to 43 patients (total number of doses: 286; median and range per patient: 8, 1‐10). Twenty‐six (60.5%) patients received the eight scheduled doses of rituximab. Rituximab maintenance was delayed in 10 of 43 (23.3%) patients due to hematological toxicity (9.3%), nonhematological toxicity (2.3%), or nonrituximab‐related toxicity (11.6%).

At least one grade 3/4 AE was observed in 53 of 60 (88.3%) patients during the induction phase and 24 of 43 (55.8%) patients during the maintenance phase. The most common grade 3/4 toxicity was hematological (Table 3), including lymphopenia (70%) and neutropenia (60%). The most frequent severe toxicities during maintenance therapy were neutropenia (27.3%) and lymphopenia (25%). Febrile neutropenia was observed in only 6.7% (4/60) of patients during induction with RBMD and 4.5% (2/43) during rituximab maintenance.

**Table 3:** Adverse events observed in >2 patients who received at least one cycle of RBMD

	Grades 1‐2		Grades 3‐4	
	RBMD (%)	R‐maintenance (%)	RBMD (%)	R‐maintenance (%)
Hematological				
Lymphopenia	12 (20)	25 (56.82)	42 (70)	11 (25)
Neutropenia	11 (18.3)	14 (31.8)	36 (60)	12 (27.3)
Anemia	38 (63.3)	17 (38.6)	1 (1.7)	1 (2.3)
Thrombocytopenia	34 (56.7)	14 (31.8)	2 (3.3)	1 (2.3)
Nonhematological				
Fatigue	13 (21.7)	2 (4.5)	1 (1.7)	
Infections	11 (18.3)	13 (27.5)	5 (8.3)^a^	4 (9.1)
Fever (nonneutropenic)	9 (15)	2 (4.5)	2 (3.3)	
Vomiting	8 (13.3)			
Infusion reactions	6 (10)		2 (3.3)	
Constipation	5 (8.3)			
Pain	5 (8.3)	4 (9.1)		
Diarrhea	4 (6.7)		1 (1.7)	
Nausea	3 (5)			
Rash	3 (5)	1 (2.3)		
Febrile neutropenia			4 (6.7)	2 (4.5)

Note

Adverse events recorded during induction and maintenance therapy in 60 patients who received at least one cycle of RBMD and were included in the toxicity analysis.

Abbreviations: RBMD, rituximab, bendamustine, mitoxantrone, and dexamethasone; R‐maintenance, rituximab maintenance therapy.

a One patient had grade 5 infection and died from septic shock due to influenza A infection.

During induction and before maintenance therapy, five (8.3%) patients were withdrawn from the trial due to a treatment cycle delay >4 weeks and three (5%) patients were drawn due to toxicity (severe infusion reaction, severe respiratory infection, and septic shock associated with respiratory infection in the context of influenza A infection after the fifth RBMD cycle, resulting in death in CR). Five (8.3%) patients developed severe infections without neutropenia and one patient (with PD during the first cycle) died. During maintenance therapy, three of 43 (7%) patients were withdrawn from the trial due to toxicity; specifically, a treatment cycle delay >4 weeks for recovery from neutropenia (n = 1); myelodysplastic syndrome (n = 1); and worsening performance status without relapse or infection (n = 1).

Granulocyte colony‐stimulating factor (G‐CSF) was administered to 34 of 60 (56.6%) patients (median, 3 doses; range, 1‐6) either as prophylaxis or as treatment during the induction phase, and to nine of 43 (20.9%) patients during rituximab maintenance (median, 1 dose; range, 1‐5).

## 5 DISCUSSION

Our results indicate that RBMD therapy using a response‐adapted strategy followed by rituximab maintenance is effective and safe in FL patients relapsed or refractory to first‐line immunochemotherapy, including those who previously received rituximab maintenance after first‐line therapy.

Treatment of patients with relapsed or refractory FL, particularly those who have received immunochemotherapy with regimens such as R‐CHOP, R‐CVP, or R‐FCM, is becoming increasingly difficult. RBMD is a new regimen for the treatment of these patients and this trial is the first specifically designed to assess this combination in a homogeneous population of FL patients previously treated with rituximab. We also evaluated the efficacy of rituximab maintenance in patients treated with regimens that included rituximab and bendamustine as salvage therapy.

The observed response rate (88.3%, including 70% CR and a median PFS of 56.4 months) is very promising, particularly given that less than 50% of patients received more than four cycles. Other regimens based on bendamustine‐containing chemotherapy plus rituximab have been previously used to treat relapsed/refractory indolent lymphoma, and have achieved response rates comparable to those of RBMD, albeit mainly in rituximab‐naïve patients. A similar schema consisting of rituximab, bendamustine, and mitoxantrone was used to treat relapsed or refractory indolent and mantle cell lymphoma patients.^19^ That study, which included 29 patients with FL (38% previously treated with rituximab), reported an ORR of 92% (CR 50%) and a median PFS of 17 months. In patients treated with BR, ORR ranged from 93% to 96% and CR rates from 54% to 71%, but the median PFS was 23‐24 months (44‐100% rituximab‐naïve).^12^, ^13^ The addition of bortezomib^25^ to BR resulted in an ORR of 88% (CR, 53%) and a median PFS of 14.9 months. In a randomized phase III trial comparing BR with FR in patients with relapsed indolent lymphoma (61% rituximab‐naïve), BR was significantly more efficacious (ORR, 82%; CR, 40%; median PFS, 34.2 months) than FR.^14^ In hard‐to‐treat rituximab‐refractory indolent lymphoma patients, bendamustine as monotherapy at a higher dose (120 mg/m^2^, days 1‐2) resulted in an ORR of 77%‐80%, although the benefit was short‐lived (median PFS, 7‐9 months),^26^, ^27^ and when combined with obinutuzumab^18^ resulted in higher minimal residual disease‐negative rates and improved efficacy and overall survival.

In recent years, novel therapies (eg, immunomodulatory therapies, PI3K inhibitors, BTK inhibitors, BLC2 inhibitors) in monotherapy or combined with rituximab have been used to treat this population. High response rates have been reported for the immunomodulator lenalidomide and for PI3K inhibitors. The Augment study of 358 patients with relapsed indolent lymphoma (84% FL; median number of previous treatments, 1) reported an ORR of 78%, CR of 34%, and a median PFS of 39.4 months after treatment with lenalidomide plus rituximab, although patients refractory to rituximab or rituximab combinations were excluded.^28^ Based on the results of phase II studies, three PI3K inhibitors (idelalisib, copanlisib, and duvelisib) are currently available for the treatment of FL that has relapsed or is refractory after two or more previous lines of therapy.^29^, ^30^, ^31^ These studies, which included heavily treated FL patients, reported ORR rates of 56%, 59%, and 42%, respectively (with respective CR rates of 14%, 14%, and 1%), and median PFS ranging from 9.5 to 11 months. One of the main concerns with PI3K inhibitors is their potential toxicity, which can cause gastrointestinal, hepatic, and infectious complications. However, their high activity makes them a therapeutic option for high‐risk FL (particularly in patients with early progression or treatment failure for whom available strategies are unsatisfactory); in these situations PI3K inhibitors may provide a bridge to transplantation.^32^ Comparatively poorer response rates have been reported for BTK inhibitors^33^ and BCL2 inhibitors.^34^, ^35^

In our study, the median PFS in the 10 patients refractory to first‐line rituximab‐containing immunochemotherapy was 25.5 months. The PFS obtained with RBMD followed by rituximab maintenance in this subgroup of patients, over half of whom received only four cycles, is similar to that reported for obinutuzumab plus bendamustine followed by obinutuzumab maintenance (25.3 months),^18^ suggesting that RBMD may be a useful alternative if obinutuzumab is unavailable.

Two recent studies proposed that POD within 2 years of diagnosis in FL patients treated with first‐line immunochemotherapy is a predictor of poor outcome.^15^, ^36^ Stratification of our patient population according to POD following first‐line therapy (before or after 24 months) revealed that the majority (n = 32, including the 10 refractory cases) corresponded to the former subgroup. We observed no significant differences in PFS between subgroups. Nonetheless, for patients who showed POD in less than 24 months the median PFS was 42.31 months, an acceptable response for patients who have a poor prognosis and are not candidates for intensive chemotherapy.

The EORTC 20891 trial demonstrated that in relapsed and refractory FL patients, rituximab maintenance improves PFS after CHOP and R‐CHOP (no patients had been previously treated with rituximab). At 6‐year follow‐up, the median PFS after induction for patients in CR was 52.8 months in the rituximab maintenance arm, vs 14.4 months in the observation arm.^10^ However, data on the efficacy of rituximab maintenance in relapsed patients who have responded to BR schedules are scarce. Currently, most patients receive rituximab maintenance after first‐line therapy. We hypothesize that after achieving a response with RBMD rescue treatment, the duration of the response can be prolonged with rituximab maintenance. Compared with other studies of bendamustine‐containing chemotherapy plus rituximab, in which the majority of patients was not treated with rituximab and did not receive rituximab maintenance therapy, our results show that rituximab maintenance improves PFS after rescue with RBMD, with no significant differences between patients who did/did not receive rituximab maintenance after first‐line therapy. The median PFS in this trial, in which all patients had been previously exposed to rituximab, was 56.4 months. This outcome is superior to those reported for bendamustine‐containing combinations without rituximab maintenance (median PFS, 17‐24 months).^12^, ^13^, ^19^ A German phase III trial that included a small subgroup of patients who received rituximab maintenance after response to BR reported a median PFS of 72.1 months, although two‐thirds of the cases were rituximab‐naïve.^14^ However, these trials included heterogeneous populations of patients, precluding accurate comparison of study outcomes. In current practice, all relapsed/refractory FL patients would have been previously exposed to rituximab during induction and/or maintenance therapy. We found no significant differences in PFS between patients who did/did not receive prior rituximab maintenance after first‐line therapy. While data on the usefulness of second rituximab maintenance are scarce,^20^ the median PFS of 42.3 months reported here suggests that second rituximab maintenance is a reasonable strategy in responding patients. In conclusion, our results show that rituximab maintenance improves PFS after rescue with RBMD, with no significant differences between patients who did/did not receive rituximab maintenance after first‐line therapy.

In our study, 55% of patients achieved CR/uCR after only three cycles of RBMD. Furthermore, two patients with PR after six RBMD cycles achieved CR during maintenance with rituximab. After rituximab maintenance, no differences in PFS were observed between the CR‐3 and CR‐6 subgroups. These results suggest that patients treated with RBMD can achieve CR with fewer cycles and identify a subset of patients who may require less rescue treatment.

Overall, dose‐adapted RBMD was well tolerated, with a safety profile similar to that reported for BR.^37^ Rituximab maintenance resulted in no unexpected toxicity. The most common toxicity during induction and maintenance was hematological (grade 3/4 neutropenia in 60% of patients), but did not translate into an increase in the number of patients with febrile neutropenia or infections. One patient developed an opportunistic infection due to CMV reactivation in the context of influenza A sepsis, and ultimately died. Fatigue was the most common nonhematological toxicity, which was tolerable. Only four cases of skin rash were recorded, likely due to the inclusion of dexamethasone in the regimen. A previous phase II trial of bendamustine and mitoxantrone plus rituximab in patients with untreated high‐risk FL was prematurely closed due to severe hematological and infectious toxicities and second malignancies.^38^ In our trial, in which all patients had received previous immunochemotherapy, we observed no severe toxicities and only one diagnosis of myelodysplastic syndrome. Our response‐adapted strategy, which limited immunochemotherapy to only four cycles in over half of all cases, together with the low dose of mitoxantrone and the early withdrawal of patients with sustained myelotoxicity, likely contributed to favorable tolerability. The use of G‐CSF and mandatory anti‐infective prophylaxis with co‐trimoxazole and acyclovir may also have made this regimen safer. Recent data from a phase III trial have raised concerns about the toxicity profile of bendamustine with rituximab or obinutuzumab followed by anti‐CD20 maintenance in first‐line treatment of FL.^39^ However, acceptable toxicity was reported in two recent phase III trials of induction therapy with BR followed by rituximab maintenance.^40^, ^41^ Additional studies with longer follow‐up periods will be required to better characterize the toxicity profile of this drug combination.

Limitations of our study include the small sample size of the refractory patient subgroup, the lack of a comparator group, and the fact that response evaluation was based on assessments made by local investigators. Moreover, some of the studies with which we have compared our findings included rituximab‐naïve patients with indolent and mantle cell lymphoma.

In summary, in patients with relapsed/refractory FL after first‐line immunochemotherapy, RBMD salvage therapy using a response‐adapted strategy followed by rituximab maintenance is effective, has an acceptable safety profile, and allows for a reduced induction therapy, thereby improving tolerability without compromising efficacy. This regimen could constitute an alternative salvage regimen for patients treated with other rituximab‐containing combinations plus rituximab maintenance in first‐line therapy.

## CONFLICT OF INTEREST

The authors declare no potential conflict of interest.

## AUTHOR CONTRIBUTIONS

Francisco‐Javier Peñalver: Study concept, study design, enrollment of patients, data collection, data analysis, study oversight, manuscript writing, and approval of final manuscript. José‐Antonio Márquez, Soledad Durán, Pilar Giraldo, Alejandro Martín, Carlos Montalbán, María‐José Ramírez, María‐José Terol, Francisco‐Javier Capote, Antonio Gutiérrez, and Blanca Sánchez: Enrollment of patients, data collection and analysis, and approval of final manuscript. Juan‐Manuel Sancho, Andrés López and Antonio Salar: Enrollment of patients, data collection, data analysis, manuscript writing, and approval of final manuscript. Gil Rodríguez‐Caravaca: data analysis, study oversight, and approval of final manuscript. Miguel Canales: Study design, enrollment of patients, data collection, data analysis, and approval of final manuscript, María‐Dolores Caballero: Study design, study oversight, funding, and approval of final manuscript.

## Data Availability

The data that support the findings of this study are available on request from the corresponding author. The data are not publicly available due to privacy or ethical restrictions.
